# Subjective Generic Health Literacy and Its Associated Factors among Adolescents: Results of a Population-Based Online Survey in Germany

**DOI:** 10.3390/ijerph17228682

**Published:** 2020-11-23

**Authors:** Anne-Kathrin M. Loer, Olga M. Domanska, Christiane Stock, Susanne Jordan

**Affiliations:** 1 Department of Epidemiology and Health Monitoring, Robert Koch Institute, General-Pape-Str. 62-66, 12101 Berlin, Germany; DomanskaO@rki.de (O.M.D.); JordanS@rki.de (S.J.); 2 Institute of Health and Nursing Science, Charité—Universitätsmedizin Berlin, Corporate Member of Freie Universität Berlin, Humboldt-Universität zu Berlin, and Berlin Institute of Health, Augustenburger Platz 1, 13353 Berlin, Germany; christiane.stock@charite.de

**Keywords:** health literacy, adolescents, MOHLAA-Questionnaire, subjective measurement, population-based survey

## Abstract

Profound data on adolescent health literacy are needed as a requirement for the development of health literacy promoting interventions. This paper aims to study the level of generic health literacy among adolescents and to explore associations between health literacy and socio-demographic (age, sex, family affluence, migration background), social (social support by family and friends) and personal (self-efficacy) factors. We conducted a representative cross-sectional online survey. Four health literacy dimensions were captured among 14–17 years old adolescents living in Germany (*n* = 1235) with the “Measurement of Health Literacy Among Adolescents-Questionnaire” (MOHLAA-Q). Descriptive, bivariate and multiple logistic regression analyses were used to analyse the data (*n* = 1202). We found poor health literacy levels—to varying degrees—in all examined health literacy dimensions: dealing with health-related information (8.41% with many difficulties), health-related communication skills (28.13% with low skills), attitudes toward one’s own health and health information (8.81% with passive attitudes) and health-related knowledge (22.73% with low levels). We identified significant associations between poor health literacy levels and all factors studied except for age. Our results indicate a need for the implementation of evidence-based health literacy-related promoting interventions, preferentially in education and training institutions.

## 1. Introduction

### 1.1. Background

Health literacy is regarded as a relevant determinant of health [1,2]. Its relation to health is clearly elaborated in the widespread definition by Sørensen et al. [3] (p. 3): “Health literacy is linked to literacy and entails people’s knowledge, motivation and competences to access, understand, appraise, and apply health information in order to make judgments and take decisions in everyday life concerning healthcare, disease prevention and health promotion to maintain or improve quality of life during the life course”. This definition regards health literacy as a multidimensional construct based on different dimensions [3]; other definitions focus on other dimensions, e.g., functional, interactive and critical health literacy [3,4]. The definition by Sørensen et al. describes health literacy in its entirety (generic health literacy), whereby other definitions concentrate on a specific area in which people use health literacy in order to make health-related decisions (specific health literacy) [5], e.g., “nutrition literacy” or “media literacy” [6]. In order to strengthen generic health literacy with positive effects on health across the life course [7,8,9,10], the promotion of health literacy in childhood and adolescence is increasingly gaining importance in public health efforts [1,8]. In order to develop effective interventions promoting health literacy among adolescents, profound knowledge on the existing levels of health literacy in this age group is necessary; however, respective data are still limited [9]. Health literacy research has so far focused on the adult population [11,12]. As the life period of adolescence has unique characteristics, vulnerabilities and social contexts, findings on adults cannot be transferred to adolescents [13]. Bröder et al. elaborated six special features for children and adolescents, which are relevant for their health literacy: disease epidemiology, demography, development, dependency, democracy und digitalization [9,14]. In terms of differential epidemiology, e.g., mid-adolescents are prone to risky behavior, such as substance misuse [15,16]. In terms of digitalization, e.g., adolescents frequently use digital information to address their health issues [17,18]. Health literacy may therefore be of particular importance as it empowers young people to actively deal with health-related issues, such as substance use, and also enables them to make their own health-related decisions and to cope with the challenge of an overabundance of invalid health-related information on the Internet [19,20].

In different countries, representative data about the level of health literacy show fairly high health literacy levels among adolescents. Sukys et al. surveyed adolescents aged 13–16 years in Lithuania, where 12.1% of the participants had a low, 70.5% a moderate and 17.4% a high health literacy level [21]. Paakkari et al. measured health literacy among adolescents in the seventh and ninth grade in Finland, where 9.3% of the participants had a low, 56.7% a moderate and 34.0% a high level [22]. In contrast to these findings, evidence suggests that health literacy levels among adolescents in Germany might be poorer: the results of a cross-sectional study based on a representative sample via population registries showed that half (47.3%) of the youngest age group (15–29 years old) had a limited health literacy level [23].

Health literacy levels are influenced by various factors. Wharf Higgins et al. propose a social ecological model, according to which intrapersonal (e.g., socio-demographic characteristics, values, and experiences) and interpersonal (e.g., social support, the influence of peer groups) factors impact on adolescents’ health literacy [24]. Regarding socio-demographic factors (intrapersonal), studies by Sukys et al. and Paakkari et al. showed higher health literacy levels among adolescents with female sex, a wealthier family and a higher education level [21,22]. In a systematic review about health literacy (functional/media dimension) and health behaviour among adolescents, however, findings for associations between health literacy and sex, but also age, were inconsistent across different studies [25]. Further studies, focusing on other study populations or health literacy constructs, affirmed the assumptions of Sukys et al. and Paakkari et al. regarding the relationship between health literacy and family wealth [25,26,27,28] and education [29,30,31]. Regarding migration background, two health literacy studies among adolescents found that migration background or its associated characteristics, such as the time of immigration, were associated with a lower health literacy level [29,32], whereas in another study no associations were found [26]. In one of the studies that found associations, however, this effect was lost when adjusting for parental education and family wealth. Regarding self-efficacy as a personal factor (intrapersonal), Ghaddar et al. (2012) examining the functional and digital health literacy among Spanish adolescents, observed that a higher sense of self-efficacy was associated with a higher health literacy level [33]. Similar associations were found by Guo et al. (2020), who compared health literacy among adolescents in Melbourne/Australia and Beijing/China in a cross-cultural comparison [34]. Social support, which can be regarded as a social factor (interpersonal), was associated with health literacy in various populations, e.g., older adults living in urban areas of China [35] or smokers with a low socio-economic status in the USA [36], but also among adolescents in Melbourne/Australia and Beijing/China [34].

Besides these study results, little is known about generic health literacy and its associated factors among adolescents [19,37,38]. Data about the levels of generic health literacy can be used to identify needs for evidence-based interventions in the general adolescent population, and data about associated factors can help tailor specific approaches to subgroups.

Some of the existing studies among adolescents are characterized by a particular focus (e.g., one specific health literacy dimension such as health-related knowledge, a specific age or ((sub-) group) [23,28,31,32,39,40,41,42]. These differences may explain the observed inconsistency of study results regarding the level of health literacy and associated factors. Evidence concerning generic health literacy based on a broad concept defining health literacy as a multidimensional construct among young people in mid-adolescence is still lacking [19,38].

Therefore, the present study “Health Literacy among Adolescents” (acronym GeKoJu) aimed to explore generic health literacy among adolescents aged 14 to 17 years in a nation-wide representative sample, while reflecting the multidimensionality of the health literacy construct by separately assessing several health literacy dimensions and associated socio-demographic, social and personal factors.

### 1.2. Objectives

This study had two objectives: The first objective was to study the levels of generic health literacy among adolescents aged 14 to 17 years, living in Germany. The second objective was to explore factors associated with having poor health literacy levels.

Based on existing evidence as outlined above, the study focuses on socio-demographic (age, sex, education, family affluence, migration background), social (social support by family and friends) and personal (self-efficacy) factors as being potentially associated with generic health literacy.

## 2. Materials and Methods

### 2.1. Study Design, Data Protection, and Ethics Approval

This cross-sectional study assessed generic health literacy among 14–17 year-old German-speaking adolescents with permanent residence in Germany. The study was performed by the Robert Koch Institute, the government’s central research institution in the field of biomedicine and public health in Germany. The study is part of the second funding period of the project “Measurement of Health Literacy Among Adolescents”—Part Two (MOHLAA 2), which is embedded in the German Health Literacy in Childhood and Adolescence (HLCA) Consortium and funded by the German Federal Ministry of Education and Research [38]. In the first funding period of the project “Measurement of Health Literacy Among Adolescents”—Part One (MOHLAA1), the self-report measurement tool “Measurement of Health Literacy Among Adolescents-Questionnaire” (MOHLAA-Q) was developed and validated for the use among adolescents aged 14–17 years [19].

The Federal Commissioner for Data Protection and Freedom of Information provided approval of the study without concern on 9 July 2019. The ethics committee at the Alice Salomon Hochschule Berlin, University of Applied Sciences (Number 06-2019/26) provided ethical approval on 8 August 2019 [38].

The research design, methodology, and methods have already been described in detail in a study protocol for this survey study [38] and another article about the development and psychometric properties of the respective health literacy measurement tool [19].

### 2.2. Recruitment, Data Collection, Sample and Weighting Procedures

The recruitment was based on two-stage stratified cluster sampling to achieve a nationally representative sample. In the first stage, a sample of 50 primary sampling units (PSUs) was selected throughout 13 federal states (“Bundesländer”) in Germany, drawn from an inventory of German communities stratified by district and according to the “BIK” classification system [38,43]. The “BIK” classification system measures the grade of urbanization, regional population density and administrative borders [43]. In the second stage, adolescents’ addresses were randomly selected from local population registers at resident registration offices [38]. The adolescents and their parents/legal guardians received invitation letters through postal mail, including study information material as well as a form for informed consent. Only after receiving a signed written informed consent by the adolescents and their parents/legal guardians, a letter containing an access code for the online survey was sent to participants [38].

Prior to participation, the adolescents and their parents/legal guardians signed a written informed consent [38].

We conducted an online survey due to the frequent internet usage—at least, almost daily—in our target population [44,45]. Data were collected from 9 September 2019 to 31 December 2019. The average time to complete the self-administered questionnaire was 28 min (SD = 12).

A total of 6608 adolescents were invited for participation. In total, 1235 adolescents (American Association for Public Opinion Research (AAPOR) response rate 4 [46]: 21.3%) took part in the survey.

To correct for deviations of the survey sample from the German population structure regarding age, sex and school type distribution, a weighting factor was applied, including design and adjustment weighting. Due to the recruitment via a two-stage stratified cluster sampling, design weighting was needed. The applied design weighting determined the selection probability of the sampling units as well as the selection probability of the participants within these units. In the adjustment weighting, age, sex, federal states of Germany (as of 31 December 2018) [47] and education [48] were extrapolated to the population data using German population statistics, and were adapted to the distribution within Germany.

### 2.3. Measures

The questionnaire covered questions on subjective generic health literacy, socio-demographic and social factors, personal and social resources, health behaviour, subjective health status, and health services use. In this study, we used the social ecological model by Wharf Higgins et al. [24] as a theoretical foundation to examine socio-demographic, social and personal factors and their association with health literacy and applied the following measures:

#### 2.3.1. Generic Health Literacy

We used the “Measurement of Health Literacy Among Adolescents-Questionnaire” (MOHLAA-Q) to assess generic health literacy [19]. The questionnaire operationalises generic health literacy as a four-dimensional construct. It reflects core dimensions of generic health literacy by assessing cognitive, behavioural, behavioural–communicative, affective and conative components [3,8,19]. The four dimensions are captured in four scales consisting of 29 items in total: Scale A is an age-adapted version of the European Health Literacy Survey Questionnaire [49] for adolescents aged 14–17 years (short name: HLS-EU-Q12-Adolescents-DE). It measures how easy or difficult adolescents perceive finding, understanding, appraising, and applying of health-related information in the domains of healthcare, prevention and health promotion. Scale B captures communication skills about health topics. Scale C focuses on health-related self-awareness, self-control, self-efficacy, motivation and interest. Scale D measures several aspects of health-related knowledge, e.g., health risks of alcohol use or patient rights. The MOHLAA-Questionnaire itself and details on the development and psychometric properties can be found elsewhere [19]. Table 1 shows an overview the of scale dimensions, component of health literacy, phrasing, response options on Likert scales, number of items, score (min–max) and category (interval). Pretest results showed varying internal consistency coefficients (Cronbach’s α) for the scales (Scale A: 0.77; Scale B: 0.59; Scale C: 0.54). More details about reliability and validity (criterion and construct validity) are provided in an article about the development of the questionnaire and its psychometric properties [19].

Mean scores were calculated for scale A, B and C, and a sum score for scale D. In scale A, one missing value within twelve items was allowed, whereas in Scale B and C no missing value was allowed. In scale D, a missing value was equated with the answer option “Do not know”, coded as “not right”. Based on the mean scores and the sum score, respectively, all scales were categorised afterwards.

The approach to developing thresholds was carried out in exchange with a researcher from the department of Survey Design and Methodology of GESIS Leibniz Institute for Social Sciences, Mannheim, Germany. The thresholds for the categorisation were defined according to their distribution in relation to various criterion variables (e.g., age, school education). The consideration of response distribution was also taken into account in the process of categorization for another health literacy instrument [22]. For this purpose, the mean scores for the scales A–C and the sum score for the scale D were first divided into intervals (e.g., 0.25, 0.5 or 1 as interval width). Then cross tabulations were used to check for the scales and the criterion variables at which threshold values the response pattern (data distribution) changed considerably, i.e., at which threshold values considerable differences in the distribution of a criterion variable were found. This process step was carried out independently by two project staff members and then compared with each other in order to rule out arbitrary category development. Cut-offs were set at these threshold values and categorised indices were formed. A further considered criterion for categorising was that the number *n* in the respective cells should not be less than 10 (preferably *n* ≥ 25, as it is often mentioned as a prerequisite for statistical analyses). The correlation between the mean scores/indices and the criterion variables was then compared with the correlation between the categorised indices and the criterion variables, using Spearman’s rank correlation coefficient and Sid ´ak adjustment to calculate significance levels. This procedure served to examine which of the categories and its numbers (e.g., two, three or five categories) led to the smallest deviation from the non-categorised distribution in order to keep the loss of information as low as possible and has been described elsewhere [26].

The single scales represent different health literacy dimensions and were analysed separately. One of the crucial arguments against an average score of all four health literacy dimensions was that the use of an average score could mask specific health literacy needs. Batterham et al. (2016) illustrated this possibility very explicitly in a comparison of two patient vignettes: using an average score, both patients would have a similar health literacy score, whereas single scores for different health literacy dimensions clearly indicated differences in health literacy needs [50]. Thus, we decided to renounce an average score and to analyse single scales, as is practiced within other measurement tools [41,51,52].

#### 2.3.2. Socio-Demographic Factors

Information on all socio-demographic factors was collected in the online survey. For participant verification, data about sex and age provided by the registration offices were cross-referenced and verified with the self-reported sex and year of birth [38]. Age in completed years (on the reference date for sampling at residents’ registration offices) was calculated based on a person’s year of birth. Participants were grouped into four age categories: 14 years, 15 years, 16 years and 17 years.

Information on education was captured with following questions: “Do you still attend school? *The vocational school is not meant here.*”, and “What kind of school do you attend? *The vocational school is not meant here.*” Open answers for type of schools were, when possible, assigned to corresponding categories with the same content. Three categories for education were created to assure the comparability of our data with population data using German population statistics (as of 31 December 2018) for weighting procedures. Adolescents who had already left school were assigned to the category “No school visit”. Adolescents, visiting a grammar school, were assigned to the category “School visit/Grammar school”. Adolescents, visiting other school types (secondary general school, intermediate school, school with secondary general school and intermediate school courses of education (connected secondary general school and intermediate school), comprehensive school (school with secondary general school, intermediate school and grammar school courses of education), specialized upper secondary school, special needs school) were assigned to the category “School visit/No grammar school”.

Adolescents’ material family affluence was assessed with the Family Affluence Scale III [53]. The measurement of family affluence as an indicator for socio-economic status is widely used in research studies [53,54] when parents/guardians as informants are not included in the study [55]. For each item, values were assigned and summed up. The sum score was divided into quintiles and categorized in low (1st quintile; <20% of the sample), middle (2nd–4th quintile; 20–80% of the sample), and high (5th quintile; >80–100% of the sample) family affluence [56].

The definition of migration background is based on the definition used in the German Health Interview and Examination Survey for Children and Adolescents (KiGGS) [57]. A one- or two-sided migration background was determined using data on the adolescent’s and the parents’ country of birth. A one-sided migration background was assumed if one parent was not born in Germany; a two-sided migration background was assumed if (1) the adolescent itself immigrated from another country and at least one parent was not born in Germany or (2) both parents were born in another country, regardless of whether the adolescent in question was born in Germany or immigrated.

#### 2.3.3. Personal Factor

We used the 10-item General Self-Efficacy Scale (GSES) to assess adolescents’ self-efficacy [58]. The scale measures the subjective belief of being able to successfully cope novel or stressful situations in life by one’s own endeavours. Participants answered the items on a 4-point Likert Scale (1 = “not true at all; 2 = “rather untrue”; 3 = “rather true”; 4 = “completely true”). The score was set to missing if more than 3 items were missing. The total sum score ranged between 7 and 100. It was interpreted as follows: the higher the sum score, the higher the sense of self-efficacy [58,59].

#### 2.3.4. Social Factors

Two of three subscales of the Multidimensional Scale of Perceived Social Support (MSPSS), namely “family” with 4 items and “friends” with 4 items, were used to determine adolescents´ perceived social support by family and peers [60,61]. The German item version was taken from the Health Behaviour in School-aged Children (HBSC) study [62]. The items were answered on a 7-point Likert scale (1 = “very strongly disagree”; 7 = “strongly agree”). For both subscales, categories for perceived support by family or friends, respectively, were generated (interval ≥0 and ≤2.9 = low; interval ≥3 and ≤5 = moderate; interval ≥5.1—max = high) [63]. Because of a small number of cases in the categories low support in both variables (perceived social support by family, *n* = 34; perceived social support by friends, *n* = 44), the categories low and moderate social support were grouped to the category low/moderate social support.

### 2.4. Statistical Analyses

The data preparation included various steps of procedure including: the creation of the final dataset under exclusion of one single participant with exclusively missing values, the execution of plausibility and consistency checks and the labeling, generation and coding of variables.

For statistical analyses, cases with at least one missing value in one of the variables were deleted; listwise, the final dataset for complete case analyses comprised 1202 participants.

Absolute unweighted frequencies, weighted percentages for categorical variables and weighted mean and linearised standard error for one metric variable (self-efficacy) were calculated to describe sample characteristics.

The distribution of levels of health literacy in its four dimensions (Scale A–D) were examined for the overall survey sample as well as cross-tabulated by socio-demographic, social and personal factors using weighted prevalence rates and 95% confidence intervals. The levels of health literacy according to self-efficacy were determined by mean estimation and 95% confidence intervals. Pearson´s chi-square tests were used to examine bivariate associations; except for the variable self-efficacy, for which linear regression models were applied.

Multiple logistic regression analyses were conducted to examine associations between socio-demographic, social and personal factors (independent variables) and poor health literacy levels (dependent variables). Therefore, health literacy scales were dichotomized based on the previously determined categories (Scale A: “Many/Some” = 1 and “Few/Barely/No = 0”; Scale B: “Low” = 1 and “Moderate/Rather high/High” = 0; Scale C: “passive/partly passive–partly active” = 1 and “Active” = 0; Scale D: “Low/Moderate” = 1 and “High” = 0). All independent variables were entered in the model at once. Odds ratios (OR) and 95% confidence intervals were reported. After the multiple logistic regression analyses, a variance inflation factor (VIF) was used to check for multicollinearity. The absence of multicollinearity was assumed if the variance inflation factor (VIF) was < 10.00. Interactions between sex and age were investigated by conducting second multiple logistic regression models for each scale, including interaction terms for age and sex. The adjusted Wald test was used to test whether interactions were significant. An F-adjusted mean residual goodness-of-fit test was applied to measure model fit. For all analyses considering associations and interactions, the level of significance was set to *p*-values < 0.05. We performed data preparation and statistical analyses using the statistic software STATA^®^ version 15.1 (StataCorp LLC, College Station, TX, USA).

## 3. Results

### 3.1. Sample Characteristics

Response proportions among age and sex were almost equally distributed. Around nine in 10 participants visited school with a higher proportion of non-grammar school pupils. Approximately a quarter of participants had a migration background and more than three quarter perceived a high social support by family and peers (Table 2).

### 3.2. Levels of Health Literacy and Associated Socio-Demographic, Social and Personal Factors—Distribution and Bivariate Analyses

#### 3.2.1. Scale A (HLS-EU-Q12-Adolescents-DE): Dealing with Health-Related Information

A few adolescents reported many, the majority reported some or few, and a few participants reported barely/no difficulties in dealing with health-related information. We found significant associations between difficulties and—except for migration background—all considered factors. The higher the family affluence, educational level, perceived social support and sense of self-efficacy, the fewer difficulties were reported. With increasing age, more adolescents stated having many or some difficulties. Girls reported having many difficulties twice as often compared with boys (Table 3).

#### 3.2.2. Scale B: Health-Related Communication Skills

Approximately, every fourth adolescent had low, nearly every third adolescent had moderate, approximately every fourth adolescent had rather high and around every tenth adolescent had high health-related communication skills. Health-related communication skills were associated with personal (self-efficacy) and social factors (social support by family and friends), but not with sociodemographic factors. Adolescents with a high perceived social support and a higher sense of self-efficacy had more pronounced health-related communication skills (Table 4).

#### 3.2.3. Scale C: Attitudes toward One’s Own Health and Health Information

We found passive attitudes toward one’s own health and health information in only a few adolescents, partly passive–partly active attitudes in more than half of the adolescents and active attitudes in nearly one in three adolescents. Education, family affluence, support by family and friends and self-efficacy were associated with attitudes toward one’s own health and health information; age, sex and migration background were not associated. With increasing family affluence and educational level, adolescents less frequently had partly passive–partly active attitudes and more often active attitudes. Adolescents with high perceived social support and higher sense of self-efficacy had more active attitudes (Table 5).

#### 3.2.4. Scale D: Health-Related Knowledge

Around every fourth participant had a low, half of the participants had a moderate and around every fourth participant had a high level of health-related knowledge. Health-related knowledge was significantly associated with age, education, family affluence, social support by friends and self-efficacy. No significant associations were found with sex, migration background and social support by family. With higher age, educational level, family affluence, perceived social support by family and friends and sense of self-efficacy, the level of knowledge increased (Table 6).

### 3.3. Associations between Poor Levels of Four Health Literacy Dimensions and Socio-Demographic, Social and Personal Factors—Multiple Logistic Regression Analyses

#### 3.3.1. Results for Single Scales

Being female, not visiting school, living in a family with low affluence and perceiving low/moderate social support by friends significantly increased the odds for experiencing many or at least some difficulties in dealing with health-related information (Scale A). Having a one-sided migration background and having a higher sense of self-efficacy significantly decreased the odds.

Adolescents living in a family with middle affluence and perceiving low/moderate social support by family and friends had significantly higher odds for having low health-related communication skills (Scale B). Adolescents of female sex and with a higher sense of self-efficacy had significantly lower odds for having low health-related communication skills.

In regard to attitudes toward one’s own health and health information (Scale C), participants not visiting school, living in a family with low or middle affluence and perceiving low/moderate support by family and friends had significantly higher odds for having passive/partly passive–partly active attitudes, whereas females and adolescents with a higher sense of self-efficacy had significantly lower odds.

Participants visiting school, but not a grammar school and living in a family with middle affluence were significantly more likely, whereas participants with a higher sense of self-efficacy were significantly less likely, to have a low or moderate level of health-related knowledge (Scale D) (Table 7).

We did not find any essential interactions for age and sex (see Supplementary Materials Table S1).

#### 3.3.2. Summarised Results for the Scales

According to the results of the multiple logistic regression analyses, age was not significantly associated with poor levels in all health literacy dimensions. On the contrary, a higher sense of self-efficacy significantly decreased the odds for having poor levels in all health literacy dimensions. In general, low or middle family affluence, a visit of a non-grammar school or no school visit and a low/moderate social support significantly increased the odds for having poor health literacy levels. Depending on the scale, female sex significantly increased or decreased the likelihood of having poor health literacy levels. A migration background was only significantly associated with difficulties in dealing with health-related information: a one-sided background decreased the odds. It is noticeable that for Scales A, B and C, more or the less the same factors were relevant for having poor health literacy levels, while for Scale D the effects differed (Table 7).

## 4. Discussion

### 4.1. Summary of Principle Findings

This study aimed to examine the levels of generic health literacy among adolescents aged 14 to 17 years living in Germany, and to explore associated socio-demographic, social and personal factors for having poor health literacy levels. The majority of the study population did not have poor health literacy levels. Hence, a minority reported many difficulties in dealing with health-related information (8.41%) and showed passive attitudes toward one’s own health and health information (8.81%), whereas a substantial proportion of the study population had low health-related communication skills (28.13%) and a low health-related knowledge level (22.73%). We also identified significant associations between poor levels across the four health literacy dimensions (dealing with health-related information, health-related communication skills, attitudes towards one’s own health and health information, health-related knowledge) and all studied socio-demographic (sex, family affluence, migration background), social (social support by family and friends) and personal (self-efficacy) factors except for age.

### 4.2. Comparison with Other Studies

#### 4.2.1. Levels of Health Literacy

We assessed difficulties in finding, understanding, appraising, and applying health-related information in the domains of healthcare, prevention and health promotion among adolescents, using Scale A, an age-adapted version of the European Health Literacy Survey Questionnaire (HLS-EU-Q12-Adolescents-DE) [49]. The results are comparable to those found in a survey that measured health literacy levels in the German adult population in a nation-wide representative sample by using a short form of the European Health Literacy Questionnaire (HLS-EU-Q16) [64]; whereas about half of the adolescents in our study (50.65%) perceived many or some difficulties in making judgements and health-related decisions, the adult study revealed that about half of the participants reported difficulties in making judgements and health-related decisions [64]. These study results hint at the possibility that difficulties already experienced in mid-adolescence may persist into adulthood.

Approximately, every fourth adolescent had low health-related communication skills. Poor health-related communication skills in our study may be explained by lower needs of adolescents to communicate about health problems due to a good overall health status in this age group [65], the decision-making in terms of health-related issues by their parents [65], or by only occasional doctor visits [66]. However, some authors suggest that adolescents perceive difficulties in addressing concerns about health-related issues to parents or doctors [67].

The vast majority of adolescents had either active (34.37%) or at least partly passive–partly active (56.82%) attitudes towards their own health. Adolescents with active attitudes show higher health-related self-awareness, self-control, self-efficacy and motivation, as well as more interest in health issues. This result is consistent with findings by Schmidt et al., who studied health-related communication, knowledge and attitudes among 9–13 years old adolescents in Western Pomerania, Germany. They found very positive attitudes, which approximately correspond with active attitudes in our study, among the majority of adolescents [41]. However, the questions measuring health-related attitudes by Schmidt et al. had a stronger focus on concrete health behaviours (e.g., importance of consumption of fruit and vegetables or tooth cleaning) than the questions of the MOHLAA-Q that focus on health-related self-awareness, self-control, self-efficacy, motivation and interest in general.

In our sample, around three quarters had a low (22.73%) or moderate (50.58%) health-related knowledge. These findings are in line with two other studies from Schmidt et al. [41] and Wallmann et al. (2011), who tested health-related knowledge among German students from different school types of around 13 years of age [42].

#### 4.2.2. The Role of Socio-Demographic, Social and Personal Factors

In contrast to two other studies measuring health literacy among adolescents with another measurement tool [21,22], age was not significantly associated with health literacy in our study when adjusted for other factors. Further studies should explore the role of different age groups in mid-adolescence for health literacy.

Female adolescents were significantly more likely to experience many or at least some difficulties in dealing with health-related information, but they were significantly less likely to have low health-related communication skills and passive/partly passive–partly active attitudes toward one’s own health and health information. In turn, with regard to health-related knowledge, no significant differences were found between boys and girls. The results regarding communication are consistent with the findings by Schmidt et al., who found that females had significantly higher communication scores compared with boys. However, in contrast to our results regarding knowledge, they found significantly higher knowledge scores in females compared with males [41].

We found significant associations between migration background and any health literacy dimension only for Scale A. In a multiple logistic regression analysis for Scale A, a one-sided migration background significantly decreased the odds for experiencing many or at least some difficulties in dealing with health-related information. This result is in contrast to other studies in which a migration background or its associated characteristics, such as the time of immigration, tended to be associated with lower health literacy levels [29,32]. However, the study of Quenzel et al. suggested that available educational and economic resources influence the level of health literacy more than migration background [32]. Our results may underline their findings, as a lower family affluence and lower educational level were associated with poor health literacy levels, while migration background was not.

A higher sense of self-efficacy was the only factor that significantly decreased the likelihood for having poor levels across all health literacy dimensions. Therefore, our findings are consistent with other study results, in which health literacy was positively associated with self-efficacy [28,68,69]. Further studies, investigating pathways between health literacy, self-efficacy and diverse health behaviours concluded that self-efficacy mediated—at least partially—the effect of health literacy on health-related behaviours [70] and self-perceived health status [68,71]. Another study found that self-efficacy had a confounding effect on health literacy’s associations with health outcomes [72].

Besides self-efficacy, social support also tends to have mediating effects between health literacy and health-related outcomes [36] or self-rated health [35] among adults. With regard to adolescents, the importance of social support for adolescents’ health literacy and health can be presumed. In a qualitative study, adolescents reported that their parents act as informants for health-related questions, as health-related decision makers and as contact partners in case of health-related problems [65]. Wharf Higgins et al. noted that parental influence can also have negative effects on their children’s health literacy by role-modeling unhealthy behaviour and creating structures in which health-decision making can be difficult. They also addressed the importance of friends in terms of communicative exchange about intimate health-related questions, e.g., substance use [24]. Our results endorse the importance of social support: low/moderate support by family and friends was significantly associated with a higher likelihood of having poor health literacy levels.

Finally, in the multiple logistic regression analyses for all four scales, the same socio-demographic, social and personal factors were included. Interestingly, for Scale C an F-adjusted mean residual goodness-of-fit test suggested that the fitted model does not adequately explain the observed variation of the scale [73]. Consequently, other factors than the ones studied by us might explain the observed results to a larger extent. For example, Schmidt et al. also examined health-related attitudes [41]. However, no other factors are considered in this study that could give an indication of which factors might create better results.

### 4.3. Strengths and Limitations of the Study

A major strength of our study was that—for the first time—nation-wide data on generic health literacy among adolescents aged 14 to 17 years living in Germany are now available. The data provided first insights into the nation-wide levels of generic health literacy and its associated factors in order to identify needs for generic health literacy promotion in the general population of adolescents and to determine approaches for evidence-based interventions.

Another strength was the very low percentage of missing values in our total sample (*n* = 1235): In Scale A, there was only 0.65%, in Scale B 0.00%, in Scale C 0.00% and in Scale D 0.08% missing values, which might be explained by the online survey method used. We used a responsive design for programming the questionnaire, so the adolescents were able to fill in the questionnaire flexibly with a variety of devices, e.g., mobile phones or notebooks. The questionnaire was also programmed in a way that the adolescents could skip questions—an option that was rarely used—and could interrupt the completion of the questionnaire in between. In addition, in paper-and-pencil surveys given answers must occasionally be set to missing values due to illegibility and ambiguity: in our online survey this handling was not necessary as the online survey was programmed in such a way that answers could be clearly assigned to answer categories. With regard to our overall results we assume that very similar results would have been generated with other survey methods, e.g., paper-and pencil surveys, as evidence suggests [74,75].

A further strength concerns our chosen analysis approach not to produce an average score, but to analyse single scales instead. Analyses of single scales for different health literacy dimensions enabled us to identify specific health literacy needs—to varying degrees—in the examined dimensions in our target group. For example, the analyses showed that depending on the health literacy dimension, sex increased or decreased the odds for having poor health literacy levels. The use of an average score may have masked this differential effect for the impact of sex on health literacy. Analysing the single scales may also have been beneficial for studying the impact of the other factors as they also tended to vary in their association with poor health literacy levels in the different health literacy dimensions.

However, evidence concerning adolescents’ participation in surveys also suggests that a personal connection to the survey topic influences the response behaviour [44]. Therefore, in our study a selection bias needs to be taken into account. Adolescents more interested in health could have been more likely to participate, which may result in an overestimation of health literacy levels.

As described in detail in the study protocol, we made efforts to increase the likelihood of participation by adolescents with a migration background by drawing more addresses in large urban cities [38]. Data of German population statistics (as of 31 December 2018) cannot be compared exactly with our data as information on migration background in population statistics is reported in a summarised format for several age groups (10–15 years; 15–20 years). Nevertheless, in these two age groups more than 30% of the young people have a migration background [76]. In comparison, in our survey 24.1% of participants had a migration background. Thus, our data collection did not fully achieve a representative sample with respect to migration background.

Another issue related to representativeness needs to be discussed: we created three categories for education in a manner that our data could be extrapolated to the population data using German population statistics (as of 31 December 2018). However, this categorisation entailed that heterogeneous groupings were collapsed together in the two categories “No school visit” and “School visit/No grammar school”. We expect that the category “No school visit” comprises adolescents with diverse school-leaving qualifications, e.g., adolescents who finished the most basic education programme in secondary general school, but also adolescents who already finished the highest school-leaving qualification (Abitur) that qualifies for university education. In addition, the category “School visit/No grammar school” includes both adolescents attending lower secondary school types and those who will prospectively pass the highest school-leaving qualification (Abitur) but are attending a comprehensive school and not a grammar school. As a result, it must be assumed that some adolescents with a higher level of education are represented in all three education categories. This may result in a bias that may lead to overestimating the levels of health literacy in our target group as evidence suggests that higher educational levels are associated with higher health literacy levels [29,30,31]. A further possible problem resulting from this misclassification bias might be that we were unable to detect existing differences in health literacy by education.

Finally, it should be mentioned that a comparison with results from other studies that use different measurement tools should be made with caution, as they may focus on different health literacy constructs [22]. Therefore, since we used a newly developed questionnaire for our survey, comparisons with other study results should be carefully interpreted.

Further discussion points, including strengths and limitations with regard to study design and sampling strategy, have already been addressed in the study protocol [38].

### 4.4. Implications for Policy and Practice

Our results reveal the presence of poor health literacy levels in all four health literacy dimensions, which calls for promoting health literacy regarding several dimensions. Future in-depth analyses could help to identify the particular components of dealing with health-related information (finding, understanding, appraising, and applying) that need to be addressed in health literacy promoting activities. Besides the promotion of dealing with health-related information, attitudes towards one’s own health and health-related knowledge, programmes should also address the promotion of health-related communication skills, because many adolescents may lack these skills in situations in which they wish to express themselves. As some evidence shows that adolescents find it easier to communicate about sensitive health-related issues by using anonymous media, e.g., via e-mail [67], a special focus should be set on creating an atmosphere in which they are able to talk about health-related issues openly. Even though it can be assumed that the promotion of one health literacy dimension would positively affect other dimensions to some extent, health literacy promoting interventions should not only address single health literacy dimensions and should, e.g., combine aspects of improving health knowledge with training in communication skills or addressing health-related attitudes, because the impact of strengthening one dimension on other dimensions may be limited [41]. However, intervention studies to promote health literacy are still lacking, as others have also pointed out [77] and therefore, the effectiveness of different approaches still needs to be studied.

Regardless of the potential pathways, our findings indicate that the enhancement of both, self-efficacy and social support—besides the promotion of health literacy—should be taken into account for the development of evidence-based health literacy-related interventions for health promotion and health care. Röthlin et al., for example, suggest that adolescents’ networks can be used effectively to pass on health advice [26]. Nevertheless, future research should investigate the effects of health promotion interventions fostering self-efficacy and social support on health literacy.

The vast majority of adolescents attend schools, extracurricular or other educational institutions. Therefore, education and training institutions are very important for the implementation of health literacy promoting interventions, as they can reach adolescents on a large and relatively non-discriminatory scope [78,79]. The strengthening of cognitive, linguistic and literal capabilities and skills—as the basis for health literacy-related decisions and actions—belongs to the core tasks of educational institutions [80]. In the “National Action Plan Health Literacy—Promoting health literacy in Germany”, a group of scientists and practitioners developed scientifically validated guidelines on how health literacy may be promoted in education and training institutions, e.g., by embedding health literacy in the curricula or by integrating project weeks focusing on health literacy [79]. As part of these actions, health-related content may be imparted. By understanding the importance of strengthened generic health literacy with positive effects on health across the life course [7,8,9,10], attitudes towards one’s own health may be positively affected. Moreover, these actions could include interactive elements, in which adolescents could share and discuss ways to adequately deal with health-related information. This exchange among students may enable adolescents to get to know the health-related problem-solving approaches of peers and support the strengthening of health-related communication skills. Recently, the project “Health-competent school: organizational development for strengthening health competence in the school setting” was launched. The project follows the recommendation of the action plan by aiming to implement the promotion of health literacy specifically in the school setting [81].

## 5. Conclusions

This paper delivers first insights into the levels of generic health literacy and its associated factors among adolescents aged 14 to 17 years living in Germany. We drew a representative sample from population registries and collected data with the MOHLAA-Q. This measurement tool was specifically developed for people in mid-adolescence based on a multidimensional construct of generic health literacy.

Our findings support the measurement of health literacy by using single scales for reflecting the multidimensionality of the health literacy construct. We found poor health literacy levels—to varying degrees—in all health literacy dimensions studied. The differentiated analysis of the levels of health literacy dimensions enabled us to identify existing specific health literacy needs in this target group, which we found especially prominent in the dimension of dealing with health-related information and health-related knowledge. With regard to the development of evidence-based health literacy-related interventions, our results indicate that interventions should address several health literacy dimensions.

## Figures and Tables

**Table 1:** Four Scales of the Measurement of Health Literacy Among Adolescents Questionnaire (MOHLAA-Q): dimension, component of health literacy, phrasing, response options on Likert Scales, number of items, score (min-max) and category (interval).

Scale	Dimension (Components of Health Literacy)	Phrasing	Response Options on Likert Scale	Number of Items	Score (Min–Max)	Category (Interval)
Scale A (HLS-EU-Q12-Adolescents-DE)	Dealing with health- related information (cognitive and behavioural)	“How easy or difficult is it for you to...?”, e.g., “… understand information on food packaging?”	1 = “very difficult” 2 = “difficult” 3 = “easy” 4 = “very easy”	12	Mean Score (1–4)	Many (≥1.0 & ≤2.5) Some (>2.5 and ≤3) Few (>3 and ≤3.5) Barely/No (>3.5 and ≤4)
Scale B	Health-related communi- cation skills(behavioural–communi- cative)	“To what extent do you agree with the following sentences?”, e.g., “It is easy for me to talk with my parents about health topics.”	1 = “strongly disagree” 2 = “somewhat disagree” 3 = “somewhat agree” 4 = “strongly agree”	4	Mean Score (1–4)	Low (≥1 and ≤2.5) Moderate (>2.5 and ≤3) Rather high (>3 and ≤3.5) High (>3.5 and ≤4)
Scale C	Attitudes towards one’s own health and health information (affective and conative)	“How much in general do you pay attention to your health?” “To what extent do you agree with the following sentences?”, e.g., “It is up to me to protect myself from diseases.”	1 = “not at all” 2 = “little” 3 = “moderate” 4 = “strong” 5 = “very strong” 1 = “strongly disagree” 2 = somewhat disagree” 3 = “neither agree or disagree” 4 = “somewhat agree” 5 = “strongly agree”	7	Mean Score (1–5)	Passive (≥1 and ≤3) Partly Passive– Partly Active (>3 and ≤4) Active (>4 and ≤5)
Scale D	Health- related knowledge (cognitive)	Question- Specific, e.g., “How does it affect the body if you regularly drink a lot of alcohol?”	single choice with 4 options (plus option “Do not know”); dichotomously coded (“right”; “not right”)	6	Sum Score (0–8)	Low (≥0 and ≤3) Moderate (≥4 and ≤5) High (≥6 and ≤8)

**Table 2:** Socio-demographic, social and personal factors among adolescents aged 14–17 (*n* = 1202) of the GeKoJu 2019 study population.

Factors	Indicator Variable	N ^1^	% ^2^
*Socio-demographic*	Age		
	14 years	325	24.36
	15 years	333	24.54
	16 years	311	25.20
	17 years	233	25.90
	Sex		
	Male	520	51.10
	Female	682	48.90
	Education		
	School visit/Grammar School	699	39.39
	School visit/No Grammar School	400	47.18
	No school visit	103	13.43
	Family Affluence		
	Low	167	16.20
	Middle	791	66.08
	High	244	17.72
	Migration background		
	No	923	75.90
	One-sided	129	10.69
	Two-sided	150	13.40
*Social*	Social support by family		
	Low/moderate	268	21.33
	High	934	78.67
	Social support by friends		
	Low/moderate	275	23.43
	High	927	76.57
*Personal*	Self-efficacy (Mean ± SD)	63.09 ± 0.53	

^1^ Unweighted number of cases. ^2^ Weighted percentages; Percentages may not add up to 100 due to rounding. Abbreviation: SD = Standard Deviation.

**Table 3:** Scale A: Weighted percentages and bivariate associations of reported difficulties in dealing with health-related information and considered factors among adolescents aged 14–17 (*n* = 1202) based on GeKoJu 2019 data.

	Many	Some	Few	Barely/No	
	% ^1^ (95% CI)	% ^1^ (95% CI)	% ^1^ (95% CI)	% ^1^ (95% CI)	*p*-value
**Total**	8.41 (6.67–10.54)	42.24 (38.84–45.72)	40.22 (36.82–43.72)	9.14 (7.18–11.56)	
*Socio-demographic*					
**Age**					0.004
14 years	9.95 (6.79–14.35)	34.85 (28.48–41.81)	47.38 (41.27–53.56)	7.83 (4.66–12.87)	
15 years	5.87 (3.53–9.61)	41.46 (35.98–47.15)	42.95 (37.55–48.52)	9.73 (6.36–14.60)	
16 years	11.88 (8.52–16.33)	40.20 (34.85–45.79)	41.26 (33.93–49.01)	6.66 (3.44–12.49)	
17 years	5.98 (3.53–9.95)	51.93 (44.64–59.13)	29.88 (24.59–35.76)	12.22 (7.58–19.11)	
**Sex**					0.002
Male	5.56 (3.44–8.85)	38.69 (33.47–44.19)	45.38 (40.62–50.23)	10.37 (7.28–14.56)	
Female	11.39 (8.94–14.39)	45.95 (40.77–51.21)	34.82 (30.29–39.64)	7.84 (5.86–10.43)	
**Education**					0.008
School visit/Grammar school	7.85 (5.81–10.53)	38.75 (35.57–42.04)	42.92 (39.58–46.37)	10.45 (7.80–13.87)	
School visit/No Grammar school	7.73 (5.39–10.97)	40.91 (35.28–46.78)	42.52 (7.09–48.13)	8.85 (6.03–12.81)	
No school visit	12.40 (8.01–18.72)	57.16 (46.15–67.49)	24.14 (17.03–33.05)	6.30 (2.39–15.55)	
**Family Affluence**					0.010
Low	12.22 (7.75–18.73)	48.63 (39.11–58.25)	35.48 (26.96–45.02)	3.68 (1.29–10.05)	
Middle	8.19 (6.14–10.85)	43.09 (39.37–46.89)	39.42 (35.22–43.79)	9.30 (6.96–12.30)	
High	5.72 (3.14–10.19)	33.23 (26.07–41.25)	47.52 (40.42–54.72)	13.53 (8.68–20.49)	
**Migration background**					0.125
No	7.96 (6.19–10.19)	41.42 (37.82–45.11)	40.96 (37.18–44.84)	9.66 (7.48–12.40)	
One-sided	9.70 (5.50–16.55)	34.86 (26.31–44.52)	45.84 (36.55–55.43)	9.59 (4.92–17.86)	
Two-sided	9.89 (6.22–15.35)	52.80 (42.33–63.02)	31.54 (22.38–42.40)	5.78 (2.34–13.54)	
*Social*					
**Social support by family**					0.000
Low/moderate	14.83 (10.19–21.08)	46.82 (10.19–21.08)	33.68 (27.31–40.71)	4.67 (2.21–9.61)	
High	6.67 (5.10–8.68)	41.00 (37.43–44.66)	41.99 (38.23–45.85)	10.35 (8.00–13.28)	
**Social support by friends**					0.000
Low/moderate	13.39 (8.90–19.65)	49.56 (43.36–55.77)	33.21 (27.93–38.95)	3.84 (2.02–7.20)	
High	6.88 (5.34–8.82)	40.00 (35.93–44.21)	42.36 (38.93–45.87)	10.76 (8.24–13.92)	
*Personal*					
**Self-efficacy** (Mean) ^2^	53.35 (49.82–56.89)	59.21 (57.70–60.72)	66.69 (65.15–68.23)	74.11 (71.04–77.18)	0.000

^1^ Weighted percentages; percentages may not add up to 100 due to rounding. ^2^ the different presentation of the variable self-efficacy is based on its metric characteristics; the variable was introduced in a separate linear regression model as the dependent variable. Abbreviation: CI = confidence interval.

**Table 4:** Scale B: Weighted percentages and bivariate associations of level of health-related communication skills and considered factors among adolescents aged 14–17 (*n* = 1202) based on GeKoJu 2019 data.

	Low	Moderate	Rather High	High	
	% ^1^ (95% CI)	% ^1^ (95% CI)	% ^1^ (95% CI)	% ^1^ (95% CI)	*p*-value
**Total**	28.13 (25.55–30.86)	33.47 (30.21–36.90)	27.75 (23.86–32.01)	10.65 (8.64–13.06)	
*Socio-demographic*					
**Age**					0.553
14 years	30.84 (25.01–37.35)	31.82 (25.95–38.33)	25.99 (19.54–33.68)	11.35 (7.97–15.92)	
15 years	23.68 (18.32–30.03)	35.29 (29.30–41.79)	30.55 (25.10–36.60)	10.48 (7.07–15.27)	
16 years	32.95 (27.14–39.32)	32.02 (24.99–39.98)	26.42 (20.74–33.00)	8.61 (5.87–12.46)	
17 years	25.11 (19.86–31.20)	34.71 (27.69–42.47)	28.05 (21.43–35.78)	12.14 (8.05–17.89)	
**Sex**					0.202
Male	30.93 (26.58–35.64)	33.64 (28.66–38.63)	26.54 (22.53–30.97)	9.07 (6.10–13.30)	
Female	25.20 (20.95–29.99)	33.48 (29.67–37.53)	29.01 (23.89–34.74)	12.30 (9.90–15.18)	
**Education**					0.253
School visit/Grammar school	26.38 (22.63–30.50)	30.67 (27.99–33.48)	31.45 (26.85–36.44)	11.51 (8.96–14.66)	
School visit/No Grammar school	30.45 (26.45–34.77)	33.49 (29.02–38.27)	25.57 (20.30–31.67)	10.49 (7.86–13.87)	
No school visit	25.12 (18.01–33.87)	41.64 (30.89–53.25)	24.54 (17.41–33.43)	8.70 (3.57–19.70)	
**Family Affluence**					0.207
Low	32.72 (25.39–41.00)	30.54 (21.53–41.33)	24.70 (17.42–33.79)	12.04 (7.40–19.00)	
Middle	29.00 (25.79–32.43)	34.25 (30.91–37.76)	26.78 (22.68–31.32)	9.97 (7.66–12.88)	
High	20.68 (15.38–27.23)	33.24 (26.79–40.39)	34.16 (26.97–42.15)	11.92 (7.77–17.87)	
**Migration background**					0.196
No	28.24 (25.15–31.55)	34.40 (31.13–37.83)	27.80 (24.86–30.95)	9.55 (7.55–12.01)	
One-sided	24.41 (16.85–33.98)	24.56 (14.33–38.78)	32.01 (20.57–46.11)	19.03 (11.44–29.95)	
Two-sided	30.45 (22.59–39.66)	35.31 (25.69–46.30)	24.05 (14.15–37.81)	10.19 (5.54–18.00)	
*Social*					
**Social support by family**					0.000
Low/moderate	47.25 (40.57–54.04)	30.05 (22.55–38.79)	18.33 (14.23–23.28)	4.37 (1.97–9.42)	
High	22.94 (20.01–26.17)	34.40 (30.71–38.29)	30.30 (25.90–35.10)	12.35 (9.92–15.29)	
**Social support by friends**					0.000
Low/moderate	45.24 (38.98–51.65)	30.13 (24.09–36.95)	19.04 (14.39–24.76)	5.59 (2.96–10.32)	
High	22.89 (20.26–25.75)	34.49 (30.87–38.30)	30.41 (25.98–35.24)	12.20 (9.70–15.23)	
*Personal*					
**Self-efficacy** (Mean) ^2^	57.91 (55.37–60.44)	61.37 (59.87–62.86)	67.15 (65.58–68.73)	71.59 (68.14–75.05)	0.000

^1^ Weighted percentages; percentages may not add up to 100 due to rounding. ^2^ the different presentation of the variable self-efficacy is based on its metric characteristics; the variable was introduced in a separate linear regression model as the dependent variable. Abbreviation: CI = confidence interval.

**Table 5:** Scale C: Weighted percentages and bivariate associations of attitudes toward one’s own health and health information and considered factors among adolescents aged 14–17 (*n* = 1202) based on GeKoJu 2019 data.

	Passive	Partly Passive– Partly Active	Active	
	% ^1^ (95% CI)	% ^1^ (95% CI)	% ^1^ (95% CI)	*p*-value
**Total**	8.81 (7.01–11.02)	56.82 (52.77–60.79)	34.37 (30.84–38.08)	
*Socio-demographic*				
**Age**				0.244
14 years	10.23 (7.14–14.45)	50.56 (44.38–56.73)	39.20 (33.38–45.35)	
15 years	9.19 (5.14–15.88)	56.27 (49.89–62.44)	34.55 (28.63–40.98)	
16 years	9.59 (6.04–14.90)	62.08 (54.57–69.09)	28.33 (22.16–35.44)	
17 years	6.35 (3.63–10.89)	58.13 (50.41–65.47)	35.52 (29.09–42.52)	
**Sex**				0.266
Male	9.05 (6.21–13.00)	59.39 (52.63–65.80)	31.57 (26.68–36.90)	
Female	8.56 (6.61–11.01)	54.15 (49.86–58.38)	37.29 (32.42–42.43)	
**Education**				0.010
School visit/Grammar school	7.05 (5.04–9.78)	53.02 (49.20–56.80)	39.94 (36.05–43.95)	
School visit/No Grammar school	10.76 (7.96–14.39)	56.65 (50.89–62.23)	32.59 (27.72–37.87)	
No school visit	7.12 (3.23–14.94)	68.62 (56.41–78.70)	24.27 (16.57–34.08)	
**Family Affluence**				0.003
Low	6.75 (4.15–10.81)	67.70 (59.69–74.79)	25.55 (18.83–33.67)	
Middle	9.61 (7.23–12.66)	56.90 (51.90–61.77)	33.48 (29.77–37.41)	
High	7.70 (3.95–14.48)	46.58 (40.68–52.58)	45.72 (38.73–52.88)	
**Migration background**				0.151
No	8.78 (6.66–11.51)	54.49 (50.24–58.67)	36.73 (33.09–40.53)	
One-sided	6.49 (3.01–13.45)	66.50 (55.51–75.91)	27.00 (19.18–36.57)	
Two-sided	10.80 (6.00–18.67)	62.34 (48.26–74.60)	26.86 (17.65–38.64)	
*Social*				
**Social support by family**				0.000
Low/moderate	20.36 (15.22–26.69)	58.48 (51.87–64.80)	21.16 (16.27–27.04)	
High	5.68 (3.93–8.13)	56.38 (51.69–60.95)	37.95 (33.82–42.26)	
**Social support by friends**				0.000
Low/moderate	15.51 (11.85–20.05)	64.14 (58.42–69.48)	20.35 (15.66–26.01)	
High	6.76 (4.93–9.19)	54.59 (50.12–58.98)	38.66 (34.78–42.69)	
*Personal*				
**Self-efficacy** (Mean) ^2^	53.74 (50.75–56.74)	61.15 (59.74–62.55)	68.69 (67.16–70.22)	0.000

^1^ Weighted percentages; percentages may not add up to 100 due to rounding. ^2^ the different presentation of the variable self-efficacy is based on its metric characteristics; the variable was introduced in a separate linear regression model as the dependent variable. Abbreviation: CI = confidence interval.

**Table 6:** Scale D: Weighted percentages and bivariate associations of level of health-related knowledge and considered factors among adolescents aged 14–17 (*n* = 1202) based on GeKoJu 2019 data.

	Low	Moderate	High	
	% ^1^ (95% CI)	% ^1^ (95% CI)	% ^1^ (95% CI)	*p*-value
**Total**	22.73 (19.15–26.75)	50.58 (47.33–53.83)	26.69 (23.13–30.58)	
*Socio-demographic*				
**Age**				0.001
14 years	29.74 (24.97–34.99)	46.69 (40.51–52.98)	23.57 (19.17–28.62)	
15 years	28.42 (21.76–36.18)	48.34 (41.16–55.59)	23.24 (17.58–30.04)	
16 years	22.92 (16.49–30.99)	49.25 (42.90–55.61)	27.81 (21.51–35.14)	
17 years	10.52 (6.40–16.82)	57.67 (49.29–65.63)	31.81 (24.03–40.76)	
**Sex**				0.619
Male	24.17 (19.39–29.70)	49.36 (44.65–54.09)	26.46 (21.75–31.78)	
Female	21.21 (16.83–26.37)	51.86 (46.84–56.84)	26.93 (22.40–31.99)	
**Education**				0.000
School visit/Grammar school	16.56 (13.24–20.50)	48.74 (45.15–52.35)	34.70 (31.36–38.20)	
School visit/No Grammar school	28.94 (23.66–34.84)	50.84 (46.52–55.15)	20.23 (15.38–26.13)	
No school visit	19.01 (12.34–28.14)	55.08 (44.48–65.25)	25.91 (15.74–39.57)	
**Family Affluence**				0.018
Low	29.74 (21.29–39.84)	48.00 (37.88–58.29)	22.26 (16.65–29.10)	
Middle	20.77 (16.93–25.21)	53.65 (49.52–57.73)	25.58 (21.64–29.96)	
High	23.61 (16.75–32.20)	41.50 (35.26–48.03)	34.88 (28.75–41.56)	
**Migration background**				0.381
No	21.60 (17.81–25.94)	50.28 (46.57–53.98)	28.12 (24.56–31.99)	
One-sided	22.19 (11.28–39.02)	53.90 (41.46–65.87)	23.91 (16.07–34.02)	
Two-sided	29.53 (20.41–40.65)	49.67 (39.71–59.64)	20.80 (14.96–28.17)	
*Social*				
**Social support by family**				0.224
Low/moderate	25.93 (19.75–33.24)	51.59 (43.98–59.13)	22.48 (17.03–29.07)	
High	21.86 (18.48–25.66)	50.31 (46.54–54.08)	27.83 (23.70–32.38)	
**Social support by friends**				0.001
Low/moderate	31.33 (24.94–38.53)	49.48 (43.11–55.86)	19.19 (13.60–26.38)	
High	20.09 (16.93–23.68)	50.92 (47.05–54.79)	28.99 (25.10–33.21)	
*Personal*				
**Self-efficacy** (Mean) ^2^	61.37 (58.89–63.85)	62.64 (61.37–63.91)	65.40 (63.57–67.24)	0.007

^1^ Weighted percentages; percentages may not add up to 100 due to rounding. ^2^ the different presentation of the variable self-efficacy is based on its metric characteristics; the variable was introduced in a separate linear regression model as the dependent variable. Abbreviation: CI = confidence interval.

**Table 7:** Odds ratios between socio-demographic, social and personal factors and poor health literacy levels among adolescents aged 14–17 (*n* = 1202) based on GeKoJu 2019 data.

	Scale A: (HLS-EU-Q12-Adolescents-DE): Difficulties in Dealing with Health-Related Information ^1^	Scale B: Health-Related Communication Skills ^2^	Scale C: Attitudes toward One’s Own Health and Health Information ^3^	Scale D: Health-Related Knowledge ^4^
*Outcome category*	Many/Some	Low	Passive/Partly Passive–Partly Active	Low/Moderate
	OR (95% CI)	OR (95% CI)	OR (95% CI)	OR (95% CI)
*Socio-demographic*				
**Age**				
14 years	Ref.	Ref.	Ref.	Ref.
15 years	1.16 (0.85–1.59)	0.65 (0.39–1.06)	1.22 (0.80–1.85)	0.99 (0.65–1.50)
16 years	1.13 (0.81–1.55)	0.97 (0.56–1.69)	1.40 (0.93–2.12)	0.77 (0.50–1.18)
17 years	1.27 (0.91–1.79)	0.70 (0.40–1.20)	0.94 (0.65–1.37)	0.66 (0.39–1.13)
**Sex**				
Male	Ref.	Ref.	Ref.	Ref.
Female	**1.56 (1.14–2.13)**	**0.63 (0.44–0.91)**	**0.65 (0.45–0.93)**	0.98 (0.71–1.35)
**Education**				
School visit/Grammar school	Ref.	Ref.	Ref.	Ref.
School visit/No Grammar school	0.98 (0.75–1.27)	1.10 (0.77–1.58)	1.19 (0.88–1.60)	**1.85 (1.32–2.60)**
No school visit	**2.08 (1.25–3.46)**	0.75 (0.45–1.25)	**1.67 (1.04–2.69)**	1.62 (0.90–2.93)
**Family Affluence**				
High	Ref.	Ref.	Ref.	Ref.
Low	**1.83 (1.04–3.21)**	1.65 (0.96–2.82)	**1.80 (1.06–3.06)**	1.32 (0.82–2.11)
Middle	1.51 (0.99–2.29)	**1.55 (1.01–2.40)**	**1.51 (1.12–2.03)**	**1.37 (1.04–1.80)**
**Migration background**				
No	Ref.	Ref.	Ref.	Ref.
One-sided	**0.67 (0.46–0.98)**	0.64 (0.37–1.10)	1.30 (0.88–1.93)	1.11 (0.73–1.68)
Two-sided	1.63 (1.00–2.66)	0.84 (0.51–1.37)	1.32 (0.69–2.54)	1.30 (0.88–1.92)
*Social*				
**Social support by family**				
High	Ref.	Ref.	Ref.	Ref.
Low/moderate	1.08 (0.78–1.50)	**2.33 (1.66–3.28)**	**1.55 (1.11–2.18)**	1.11 (0.73–1.69)
**Social support by friends**				
High	Ref.	Ref.	Ref.	Ref.
Low/moderate	**1.58 (1.18–2.11)**	**1.98 (1.43–2.74)**	**1.71 (1.14–2.57)**	1.54 (0.97–2.43)
*Personal*				
**Self-efficacy**	**0.95 (0.94–0.96)**	**0.97 (0.95–0.98)**	**0.96 (0.94–0.97)**	**0.99 (0.98–1.00)**

^1^ F-adjusted test statistic = F(9.41) = 0.914; *p* = 0.522. ^2^ F-adjusted test statistic = F(9.41) = 0.667; *p* = 0.733. ^3^ F-adjusted test statistic = F(9.41) = 2.216; *p* = 0.041. ^4^ F-adjusted test statistic = F(9.41) = 1.101; *p* = 0.384. Boldface indicates *p* < 0.05. Abbreviations: CI = confidence interval; OR = odds ratio; Ref. = reference category.

## Supplementary Materials

The following are available online at https://www.mdpi.com/1660-4601/17/22/8682/s1, Table S1: Odds ratios between socio-demographic, social and personal factors and poor health literacy levels among adolescents aged 14–17 (*n* = 1202) based on GeKoJu 2019 data: models with and without interactions between age and sex.
