# Correction: Loer et al. Subjective Generic Health Literacy and Its Associated Factors among Adolescents: Results of a Population-Based Online Survey in Germany. *Int. J. Environ. Res. Public Health* 2020, *17*, 8682

**DOI:** 10.3390/ijerph19031848

**Published:** 2022-02-07

**Authors:** Anne-Kathrin M. Loer, Olga M. Domanska, Christiane Stock, Susanne Jordan

**Affiliations:** 1 Department of Epidemiology and Health Monitoring, Robert Koch Institute, General-Pape-Str. 62-66, 12101 Berlin, Germany; DomanskaO@rki.de (O.M.D.); JordanS@rki.de (S.J.); 2 Institute of Health and Nursing Science, Charité—Universitätsmedizin Berlin, Corporate Member of Freie Universität Berlin, Humboldt-Universität zu Berlin, and Berlin Institute of Health, Augustenburger Platz 1, 13353 Berlin, Germany; christiane.stock@charite.de

## 1. Incorrect Reference/Typing Error

In the discussion chapter (page 15 ff.) of the original paper [1], the numbering of some of the source references slipped or was incorrect, due to incorrect links caused by the literature management program used. The revised references are in ascending order listed below, at the end of this correction paper. Please note that the references before the discussion section in the original paper are correct.

Reference [36] on page 15 was written as: Stewart et al., reference [2]. It should have been: Sørensen et al., reference [3].

Reference [50] on page 15 was written as: Batterham et al., reference [4]. It should have been: Harvey et al., reference [5].

Reference [15] on page 16 was written as: Chung et al., reference [6]. It should have been: Schmidt et al., reference [7].

Reference [55] on page 16 was written as: Torsheim et al., reference [8]. It should have been: Domanska et al., reference [9].

Reference [58] on page 16 was written as: Schwarzer, reference [10]. It should have been: Archer and Lemeshow, reference [11]. 

Reference [31] on page 16 was written as: Richter et al., reference [12]. It should have been: Schmidt et al., reference [7].

Reference [53] on page 18 was written as: Hartley et al., reference [13]. It should have been: Harvey et al., reference [5].

Reference [31] in the chapter “4.4. Implications for Policy and Practice” on page 18 was written as: Richter et al., reference [12]. It should have been: Schmidt et al., reference [7].

Reference [15] on page 18 was written as: Chung et al., reference [6]. It should have been: Röthlin et al., reference [14].

Reference [61] on page 18 was written as: Zimet et al., reference [15]. It should have been: Geene and Rosenbrock, reference [16].

Reference [62] on page 18 was written as: Currie et al., reference [17]. It should have been: Schaeffer et al., reference [18].

Reference [63] on page 18 was written as: Zimet, reference [19]. It should have been: Hurrelmann et al., reference [20].

Reference [5] on page 18 was written as: O Neill et al., reference [21]. It should have been: Bröder et al., reference [22].

Reference [6] on page 18 was written as: Truman et al., reference [23]. It should have been the reference by Bundesministerium für Familie, Senioren, Frauen und Jugend [24].

Reference [64] on page 19 was written as: Jordan and Hoebel, reference [25]. It should have been: Bauer, reference [26].

In Table 1, there was a typing error. The number “1” in the sum score for scale D has to be replaced by the number “0”.

The authors apologize for any inconvenience caused and state that the scientific conclusions are unaffected. The original article has been updated.

## References

[1] Loer A.-K.M., Domanska O.M., Stock C., Jordan S. (2020). Subjective Generic Health Literacy and Its Associated Factors among Adolescents: Results of a Population-Based Online Survey in Germany. Int. J. Environ. Res. Public Health.

[2] Stewart D.W., Reitzel L.R., Correa-Fernández V., Cano M., Adams C.E., Cao Y., Li Y., Waters A.J., Wetter D.W., Vidrine J.I. (2014). Social support mediates the association of health literacy and depression among racially/ethnically diverse smokers with low socioeconomic status. J. Behav. Med..

[3] Sørensen K., Van den Broucke S., Pelikan J.M., Fullam J., Doyle G., Slonska Z., Kondilis B., Stoffels V., Osborne R.H., Brand H. (2013). Measuring health literacy in populations: Illuminating the design and development process of the European Health Literacy Survey Questionnaire (HLS-EU-Q). BMC Public Health.

[4] Batterham R.W., Hawkins M., Collins P.A., Buchbinder R., Osborne R.H. (2016). Health literacy: Applying current concepts to improve health services and reduce health inequalities. Public Health.

[5] Harvey K., Churchill D., Crawford P., Brown B., Mullany L., Macfarlane A., McPherson A. (2008). Health communication and adolescents: What do their emails tell us?. Fam. Pract..

[6] Chung T., Creswell K.G., Bachrach R., Clark D.B., Martin C.S. (2018). Adolescent binge drinking. Alcohol Res..

[7] Schmidt C.O., Fahland R.A., Franze M., Splieth C., Thyrian J.R., Plachta-Danielzik S., Hoffmann W., Kohlmann T. (2010). Health-related behaviour, knowledge, attitudes, communication and social status in school children in Eastern Germany. Health Educ. Res..

[8] Torsheim T., Cavallo F., Levin K.A., Schnohr C., Mazur J., Niclasen B., Currie C. (2016). Psychometric validation of the revised Family Affluence Scale: A latent variable approach. Child. Indic. Res..

[9] Domanska O., Firnges C., Bollweg T.M., Sørensen K., Holmberg C., Jordan S. (2018). Do adolescents understand the items of the European Health Literacy Survey Questionnaire (HLS-EU-Q47)—German version? Findings from cognitive interviews of the project “Measurement of Health Literacy Among Adolescents” (MOHLAA) in Germany. Arch. Public Health.

[10] Schwarzer R.J.M. (1995). Measures in health psychology: A user’s portfolio. Causal Control Beliefs.

[11] Archer K.R., Lemeshow S. (2006). Goodness-of-fit test for a logistic regression model fitted using survey sample data. Stata J..

[12] Richter D., Mehnert A., Forstmeyer D., Ernst J., Geue K. (2019). Health literacy in adolescent and young adult cancer patients and its association with health outcomes. J. Adolesc. Young Adult Oncol..

[13] Hartley J.E., Levin K., Currie C. (2016). A new version of the HBSC Family Affluence Scale—FAS III: Scottish qualitative findings from the international FAS development study. Child. Indic. Res..

[14] Röthlin F., Pelikan J.M., Ganahl K. (2013). Austrian health literacy among 15-year-old adolescents. Final Report of the Austrian Health Competence Youth Study Commissioned by the Hauptverband der Österreichischen Sozialversicherungsträger (HVSV).

[15] Zimet G.D., Powell S.S., Farley G.K., Werkman S., Berkoff K.A. (1990). Psychometric characteristics of the Multidimensional Scale of Perceived Social Support. J. Pers. Assess..

[16] Geene R., Rosenbrock R., Gold C., Lehmann F. (2012). The setting approach in health promotion with children and adolescents. [Der Settingansatz in der Gesundheitsförderung mit Kindern und Jugendlichen]. Gesundes Aufwachsen für Alle! Anregungen und Handlungshinweise für die Gesundheitsförderung bei Sozial Benachteiligten Kindern, Jugendlichen und Ihren Familien.

[17] Currie C., Inchley J., Molcho M., Lenzi M., Veselska Z., Wild F. (2014). Health Behaviour in School-Aged Children (HBSC) Study Protocol: Background, Methodology and Mandatory Items for the 2013/14 Survey.

[18] Schaeffer D., Hurrelmann K., Bauer U., Kolpatzik K.H. (2018). National Action Plan Health Literacy. Promoting Health Literacy in Germany. [Nationaler Aktionsplan Gesundheitskompetenz. Die Gesundheitskompetenz in Deutschland stärken].

[19] Zimet G. Multidimensional Scale of Perceived Social Support (MSPSS)-Scale Items and Scoring Information. https://www.researchgate.net/publication/311534896_Multidimensional_Scale_of_Perceived_Social_Support_MSPSS_-_Scale_Items_and_Scoring_Information.

[20] Hurrelmann K., Bauer U., Okan O. (2018). Strenghtening health literacy in the education sector. [Stärkung der Gesundheitskompetenz im Bildungssektor.]. Monit. Versorg..

[21] O Neill B., Gonçalves D., Ricci-Cabello I., Ziebland S., Valderas J. (2014). An overview of self-administered health literacy instruments. PLoS ONE.

[22] Bröder J., Okan O., Bauer U., Schlupp S., Pinheiro P. (2019). Advancing perspectives on health literacy in childhood and youth. Health Promot. Int..

[23] Truman E., Bischoff M., Elliott C. (2019). Which literacy for health promotion: Health, food, nutrition or media?. Health Promot. Int..

[24] Bundesministerium für Familie, Senioren, Frauen und Jugend In Joint Responsibility: Policy for, with and by Youth. The Federal Government’s Youth Strategy. https://www.bmfsfj.de/blob/141940/bfd79e3fc3acf5197251512ccec1d901/in-gemeinsamer-verantwortung-politik-fuer-mit-und-von-jugend-data.pdf.

[25] Jordan S., Hoebel J. (2015). Health literacy of adults in Germany: Findings from the German Health Update (GEDA) study. Bundesgesundheitsbl. Gesundh. Gesundh..

[26] Bauer U. GeKoOrg-Schule. Gesundheitskompetente Organisation Schule. https://gekoorg-schule.de/ueber-gekoorg/.

