# Supplementary material for: Subjective Generic Health Literacy and Its Associated Factors among Adolescents: Results of a Population-Based Online Survey in Germany
Source: Int J Environ Res Public Health. 2020 Nov 23;17(22):8682. doi: 10.3390/ijerph17228682 (PMC7709029; doi:10.3390/ijerph17228682)
Supplement: Supplementary file 1 [file ijerph-17-08682-s001.pdf]

Table S1 Odds ratios between socio-demographic, social and personal factors and poor health literacy levels among adolescents aged 14-17 (*n* = 1202) based on GeKoJu 2019 data: models with and without interactions between age and sex.

	**Scale A**		**Scale B**		**Scale C**		**Scale D**	
	model without interactions	*model with interactions*	*model without interactions*	*model with interactions*	*model without interactions*	*model with interactions*	*model without interactions*	*model with interactions*
Outcome category	Many/Some		Low		Passive/ Partly passive-partly active		Low/moderate	
	OR (95% CI)	OR (95% CI)	OR (95% CI)	OR (95% CI)	OR (95% CI)	OR (95% CI)	OR (95% CI)	OR (95% CI)
Socio-demographic								
**Age**								
14 years	Ref.	Ref.	Ref.	Ref.	Ref.	Ref.	Ref.	Ref.
15 years	1.16	1.3	0.65	0.78	1.22	1.35	0.99	1.13
	(0.85-1.59)	(0.79-2.12)	(0.39-1.06)	(0.39-1.56)	(0.80-1.85)	(0.65-2.79)	(0.65-1.50)	(0.58-2.21)
16 years	1.13	0.95	0.97	1.19	1.4	1.46	0.77	0.71
	(0.81-1.55)	(0.52-1.75)	(0.56-1.69)	(0.54-2.58)	(0.93-2.12)	(0.80-2.68)	(0.50-1.18)	(0.38-1.31)
17 years	1.27	1.48	0.7	0.97	0.94	1.47	0.66	0.61
	(0.91-1.79)	(0.89-2.45)	(0.40-1.20)	(0.49-1.93)	(0.65-1.37)	(0.81-2.67)	(0.39-1.13)	(0.29-1.29)
**Sex**								
Male	Ref.	Ref.	Ref.	Ref.	Ref.	Ref.	Ref.	Ref.
Female	1.56**	1.63	0.63*	0.91	0.65*	0.86	0.98	0.94
	(1.14-2.13)	(0.99-2.67)	(0.44-0.91)	(0.47-1.76)	(0.45-0.93)	(0.49-1.53)	(0.71-1.35)	(0.57-1.55)
**Education**								
School visit/Grammar school	Ref.	Ref.	Ref.	Ref.	Ref.	Ref.	Ref.	Ref.
School visit/No Grammar school	0.98	0.98	1.1	1.1	1.19	1.2	1.85***	1.85***
	(0.75-1.27)	(0.75-1.28)	(0.77-1.58)	(0.76-1.58)	(0.88-1.60)	(0.89-1.63)	(1.32-2.60)	(1.31-2.61)
No school visit	2.08**	2.08**	0.75	0.75	1.67*	1.65*	1.62	1.64
	(1.25-3.46)	(1.27-3.42)	(0.45-1.25)	(0.45-1.24)	(1.04-2.69)	(1.05-2.60)	(0.90-2.93)	(0.91-2.95)

**Family Affluence**								
High	Ref.	Ref.	Ref.	Ref.	Ref.	Ref.	Ref.	Ref.
Low	1.83*	1.78*	1.65	1.63	1.80*	1.77*	1.32	1.3
	(1.04-3.21)	(1.02-3.12)	(0.96-2.82)	(0.95-2.77)	(1.06-3.06)	(1.03-3.03)	(0.82-2.11)	(0.81-2.10)
Middle	1.51	1.46	1.55*	1.54	1.51**	1.47*	1.37*	1.36*
	(0.99-2.29)	(0.96-2.23)	(1.01-2.40)	(1.00-2.38)	(1.12-2.03)	(1.08-2.01)	(1.04-1.80)	(1.02-1.82)
**Migration background**								
No	Ref.	Ref.	Ref.	Ref.	Ref.	Ref.	Ref.	Ref.
One-sided	0.67*	0.67*	0.64	0.65	1.3	1.32	1.11	1.11
	(0.46-0.98)	(0.45-0.98)	(0.37-1.10)	(0.37-1.11)	(0.88-1.93)	(0.89-1.95)	(0.73-1.68)	(0.73-1.69)
Two-sided	1.63	1.62	0.84	0.85	1.32	1.35	1.3	1.29
	(1.00-2.66)	(0.99-2.66)	(0.51-1.37)	(0.53-1.39)	(0.69-2.54)	(0.71-2.56)	(0.88-1.92)	(0.87-1.90)
Social								
**Social support by family**								
High	Ref.	Ref.	Ref.	Ref.	Ref.	Ref.	Ref.	Ref.
low/moderate	1.08	1.07	2.33***	2.31***	1.55*	1.54*	1.11	1.1
	(0.78-1.50)	(0.78-1.48)	(1.66-3.28)	(1.64-3.24)	(1.11-2.18)	(1.09-2.17)	(0.73-1.69)	(0.72-1.69)
**Social support by friends**								
High	Ref.	Ref.	Ref.	Ref.	Ref.	Ref.	Ref.	Ref.
low/moderate	1.58**	1.61**	1.98***	1.99***	1.71*	1.73**	1.54	1.54
	(1.18-2.11)	(1.20-2.14)	(1.43-2.74)	(1.43-2.75)	(1.14-2.57)	(1.15-2.59)	(0.97-2.43)	(0.98-2.43)
Personal								
**Self-efficacy**	0.95***	0.95***	0.97***	0.97***	0.96***	0.96***	0.99**	0.99**
	(0.94-0.96)	(0.94-0.96)	(0.95-0.98)	(0.95-0.98)	(0.94-0.97)	(0.94-0.97)	(0.98-1.00)	(0.98-1.00)
**Interactions age & sex**								
14 years*male		Ref.		Ref.		Ref.		Ref.
15 years*female		0.81		0.69		0.82		0.77
		(0.44-1.50)		(0.29-1.62)		(0.31-2.13)		(0.34-1.75)
16 years *female		1.43		0.67		0.93		1.18
		(0.67-3.04)		(0.26-1.74)		(0.42-2.09)		(0.57-2.42)
17 years *female		0.74		0.48		0.42		1.21

	(0.38-1.43)	(0.18-1.31)	(0.18-1.02)	(0.55-2.67)
Adjusted Wald test statistic, exact p-value	F(3.47) = 0.84 *p* = 0.48	F(3.47) = 0.70 *p* = 0.56	F(3.47) = 1.55 *p* = 0.21	F(3.47) = 0.43 *p* = 0.73

* $p < 0.05$; ** $p < 0.01$; *** $p < 0.001$

Abbreviations: CI = confidence interval; OR = odds ratio; Ref. = reference category.
